# Reconstruction of the Tongue after Hemiglossectomy Using Serratus Anterior Muscle Free Flap

**DOI:** 10.1155/2023/6637271

**Published:** 2023-10-09

**Authors:** Petr Šín, Alica Hokynková, Pavel Rotschein, Radek Pejčoch, Lucie Nártová

**Affiliations:** ^1^Department of Burns and Plastic Surgery, University Hospital Brno, Czech Republic Jihlavská 20, 625 00 Brno, Czech Republic; ^2^Faculty of Medicine, Masaryk University Brno, Czech Republic Kamenice 5, 625 00 Brno, Czech Republic; ^3^Clinic of Pediatric Surgery, Orthopedics and Traumatology, University Hospital Brno, Czech Republic Jihlavská 20, 625 00 Brno, Czech Republic; ^4^Department of Otorhinolaryngology, University Hospital Brno, Czech Republic Jihlavská 20, 625 00 Brno, Czech Republic

## Abstract

**Background:**

Serratus anterior muscle free flap is widely used in numerous indicated reconstructions. Only a few studies have dealt with the use of this flap in tongue reconstruction.

**Materials and Methods:**

We present a case series of 7 patients with carcinoma of the tongue who underwent hemiglossectomy followed by immediate reconstruction with serratus anterior muscle free flap between January 2017 and December 2019 at the University Hospital Brno. The aim of this study was to evaluate safety and efficiency of the reconstruction as well as the donor site morbidity.

**Results:**

There was not a single case of flap failure observed and the donor site healed completely in all cases. The functional outcome (tongue mobility, phonation, and deglutition) depended on the severity of the primary oncological disease and health status of the patient.

**Conclusion:**

The serratus anterior muscle free flap represents an alternative option for reconstruction of the tongue.

## 1. Introduction

Growing incidence of tongue tumours places higher demands on its reconstruction [1]. In order to ensure good functional result and proper tongue function after hemiglossectomy, an adequate volume of the missing mass and shape must be restored. Free flap transfer is the current method of choice in tongue reconstruction with radial forearm free flap (RFFF) and anterolateral thigh free flap (ALTFF) considered as workhorses [2].

The serratus anterior muscle free flap (SAMFF) provides a thin and pliable muscle tissue, which is easy to harvest and has long vascular pedicle of good calibre. There are only some cases of tongue reconstruction with SAMFF described in the literature despite its advantages.

In this case series, we evaluated tongue reconstruction using SAMFF in 7 patients who underwent hemiglossectomy at the University Hospital Brno. The aim of the study was to evaluate safety and efficiency of the reconstruction, functional result of the restored tongue, eventual complications of wound healing, or flap failure and donor site morbidity.

## 2. Case Series

We present a case series of 7 patients who underwent hemiglossectomy for various carcinomas of the tongue between January 2017 and December 2019 at the University Hospital Brno. Inclusion criteria were surgical resectability of the tumour and the need of free flap reconstruction. The surgical resection was followed by an immediate reconstruction of the tongue with muscle flap SAMFF in all the patients (no myocutaneous form was performed). The radical tumour resection with or without laryngeal preservation, alongside with deep cervical lymphadenectomy for an advanced stage of invasive tumour, was performed by otolaryngologists, and the immediate reconstruction using SAMFF was performed by plastic surgeons as a one stage procedure. The design of the flap was adapted to the missing volume and shape, and an oval shape was used in all cases. The flap was spread and modelled at the site of the missing tissue to achieve maximum symmetry with the unaffected side of the tongue.

There were 5 female and 2 male patients in the series with the average age of 63.8 years (48–82 years). Following parameters were assessed: age, sex, tumour characteristics, histopathological classification, complications in healing, and flap failure (Table 1). Functional outcomes such as phonation and oropharyngeal dysphagia evaluated by flexible endoscopic evaluation of swallowing (FEES) were followed by an attending ENT surgeon. Morbidity of donor site (pain, scar, and shoulder mobility) was evaluated by an attending plastic surgeon.

**Figure 1:**
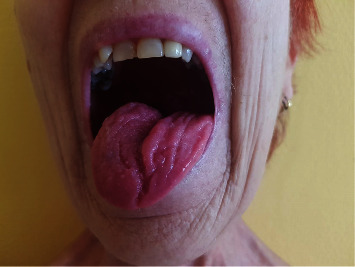
Reconstructed tongue using MSAFF, 26 months after the surgery.

**Figure 2:**
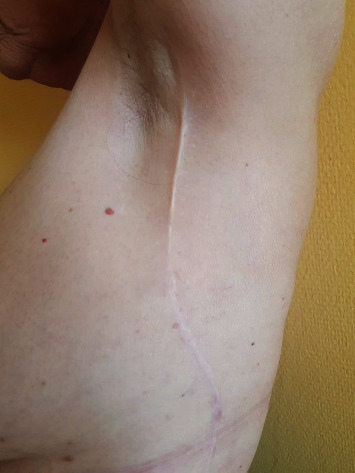
Low donor site morbidity after harvesting MSAFF, 26 months after the surgery.

There was not a single case of flap loss observed in the postoperative period. There were only minor complications in wound healing in two cases of the reconstructed tongue which were treated conservatively. The healing of the flaps in the oral cavity was not accompanied by any complications despite the exposure of saliva, and a very good aesthetic result was achieved after the resolution of the swelling and the maturation of the tissues. The functional outcome of the reconstructed tongue (Figure 1) depended on the severity of the primary oncological disease and the health status of the patient. The donor site healed completely in all cases, and no functional disorders of the upper extremity movement were observed (Figure 2). In majority of patients, the results of the tongue mobility, phonation, and deglutination were evaluated as adequate.

## 3. Discussion

Free flap transfer represents a method of choice in tongue reconstruction after hemiglossectomy. Fasciocutaneous and perforator free flaps such as RFFF and ALTFF are the method of choice. Muscle free flaps, such as rectus abdominis muscle free flap [3, 4], vastus lateralis myofascial free flap [5], or gracilis muscle free flap [6], are used rather rarely. The myocutaneous form of SAMFF was used by Janik et al. for reconstruction in 7 patients who underwent salvage glossectomy [7].

SAMFF in general is a highly reliable flap with excellent anatomical characteristics for free flap reconstruction of small and moderate defects. It is easy to harvest and well-suited for head and neck reconstruction.

The main disadvantages of this technique include the impossibility to use the two-team approach and donor site morbidity with the loss of the muscle tissue. In our study, we did not observe any problems with the upper extremity function, wound healing, or resulting scar in the postoperative follow-up. The scar was well accepted by the patients. Second, it is not possible to assess directly the flap perfusion of MSAFF by capillary refill, as it can be performed in case of fasciocutaneous or perforator flaps. On the other hand, capillary refill assessment of fasciocutaneous or perforator flaps used on reconstruction of deeply localized defects of tongue base is compromised by perioperative and postoperative swelling. Standard examination of muscle flap is performed by the Doppler probe, which identifies vascular pedicle pulsation. The microsutures are often performed on recipient neck vessels. Therefore, high interference from surrounding vessels prevents detection of vascular pedicle of muscle flap by dopplerometry.

## 4. Conclusion

The SAMFF represents an alternative option for tongue reconstruction after hemiglossectomy. The main advantage of this flap is muscle-to-muscle connection, natural look of the reconstructed tongue, and easy harvesting of the flap with low donor site morbidity.

## Figures and Tables

**Table 1:** Study participants, surgical treatment, and functional outcomes.

Age/sex	Smoking	Type of tumour	Recurrence	Preoperative actinotherapy	TNM classification	Lymphatic nodes metastases	Neck dissection	Laryngeal preservation	Total surgery time/min	Flap failure	FEES-oropharyngealdysfunction
48/F	Stop	Adenocarcinoma	No	No	pT4N0	0/7 LN (+ perineural invasion)	Bilateral	Yes	570	No, revision for small abscess in remaining tongue tissue	Medium severity
60/F	Yes	Spinocellular carcinoma (G3)	Yes	Yes	pT3N0	0/2 LN (+ perineural invasion)	Unilateral -l. dx.	No, supraglottic laryngectomy performed	360	No, completely healed	High severity
61/M	Stop	Spinocellular carcinoma (G3)	Yes	Yes	pT3N1	1/4 LN (+ perineural invasion)	Unilateral -l. sin.	Yes	555	No, marginal necrosis	Not performed
64/M	Yes	Spinocellular carcinoma (G2)	Yes	Yes	pT3N3b	9/9 LN (+ perineural invasion)	Unilateral -l. dx.	Yes	495	No, completely healed	High severity
65/F	No	Spinocellular carcinoma (G3)	No	No	pT2N1	1/6LN (+ perineural invasion)	Bilateral	Yes	495	No, small wound dehiscence (up to 1 cm)	Mild severity
67/F	Yes	Mucoepidermal carcinoma	No	No	pT3N0	0/3 LN (+ perineural invasion)	Unilateral -l. dx.	Yes	420	No, completely healed	Medium to high severity
82/F	Non-	Spinocellular carcinoma (G1)	Yes	No	pT3N0	0/8 LN (0 perineural invasion)	Unilateral -l. dx.	Yes	450	No, completely healed	Mild to medium severity

## Data Availability

No data were used in this study.
